# Chinese Children’s Knowledge of Topicalization: Experimental Evidence from a Comprehension Study

**DOI:** 10.1007/s10936-018-9575-6

**Published:** 2018-03-30

**Authors:** Shenai Hu, Maria Teresa Guasti, Anna Gavarró

**Affiliations:** 1 Department of Foreign Language Education, Xiamen University, Xiamen, 361005 China; 2 Departament de Filologia Catalana, Universitat Autònoma de Barcelona, 08193 Bellaterra, Spain; 3 Department of Psychology, University of Milano-Bicocca, 20126 Milan, Italy

**Keywords:** Chinese topicalization, Topic marker, Child acquisition, Movement analysis, Base-generation analysis

## Abstract

There is a debate as to whether topic structures in Chinese involve A’-movement or result from base-generation of the topic in the left periphery. If Chinese topicalization was derived by movement, under the assumptions of Friedmann et al.’s Relativized Minimality (Lingua 119:67–88, 2009), we would expect children’s comprehension of object topicalization (with OSV order) to be worse than their comprehension of subject topicalization (with SVO order). This study examined 146 Mandarin-speaking children from age three to age six by means of a picture-sentence matching task with an appropriate context. The results showed a subject/object asymmetry when the topic marker is overt, and no asymmetry when the topic marker is covert. This suggests that the presence or absence of topic markers play an important role in children’s comprehension of topicalization. We propose that both structures involve movement in the adult grammar, but not in the child grammar, at least initially. Sentences without overt topic markers are base-generated on a par with gapless sentences with a topic, and the base-generation analysis is abandoned as soon as children learn the syntax and semantics of topic markers, which function as attractors of topics.

## Introduction

Chinese has been claimed to be a topic-prominent language, distinguishing itself from many other subject-prominent languages, such as English (Li and Thompson 1976, 1981). Topics in Chinese are marked by their sentence-initial position, optionally followed by overt topic markers such as *ya*. The topic structure has been widely discussed in the linguistic literature and authors have critically discussed whether it is derived by movement or not (Chao 1968; Huang et al. 2009; Li 2000; Li and Thompson 1976, 1981; Xu 2000; Xu and Langendoen 1985; amongst others). However, not much is known on how children acquire this structure during development, except for a few studies focusing on the children’s spontaneous speech (Chen 2009; Erbaugh 1992). In this article, we attempt to provide a picture of the comprehension of the Chinese topic structure, by focusing on the comparison between subject topicalization (with SVO order) and object topicalization (with OSV order), and by examining topicalization with and without an overt topic marker. This allows us to approach the Chinese topic structure from the acquisition perspective.

We organize the article as follows. We first discuss two contrasting approaches to Chinese topic structures, one arguing for movement and the other for base-generation, and briefly review previous acquisition studies on Chinese topic structures. Then we provide the movement versus non-movement analyses of topicalization and discuss their predictions for acquisition within the Relativized Minimality framework (Friedmann et al. 2009). After that we present the details of two experiments and offer a discussion.

### A Debated Issue: Does Chinese Topicalization Involve Movement or Not?

Chinese topic structures include three elements: a topic, a comment and a topic marker. A topic is typically a nominal, referring either to a specific entity (that the hearer already knows) or a class of entities, but other syntactic categories can also constitute the topic. Generally, the comment is a clause, but not obligatorily. The topic marker can be null as in (1a), or overt like *ya* in (1b). Not only *ya*, but also *a*, *me*, *ne* and *ba* can be used as topic markers (Li and Thompson 1981).

**Table:** 

(1)	a.	Li	xiansheng_i_,	wo	renshi	e_i_.	
		Li	Mr.	I	know		
	b.	Li	xiansheng_i_	ya,	wo	renshi	e_i_.
		Li	Mr.	TOP	I	know	
		‘As for Mr. Li, I know (him).’					

A comma is often placed after a topic or topic marker in the written language, but this does not mean that a pause is required in the spoken language (Xu 2000). The use of overt topic markers, the same as the pause, is optional and largely depends on individual speakers (Li and Thompson 1981; Xu 2000). However, the discourse pragmatic roles of each topic marker are not the same (Chu 2003, 2006; Lee 2003; Yuan 2003; Zhang and Fang 1996). According to Chu’s (2003) investigation, *a*/*ya* are almost equal to a simple pause and their function is simply to signal a topic, without any additional meaning. *Ba* often follows an aforementioned event and *ma* often follows an aforementioned object, while *ne* sometimes signals a contrast between the marked topic and another topic or a juxtaposition of two topics (Chu 2003). In this regard, the use of overt topic markers is not optional, as they are of different pragmatic import.

Consider the topic structures in (2), which, together with (1), illustrate the variation encountered in topic structures.
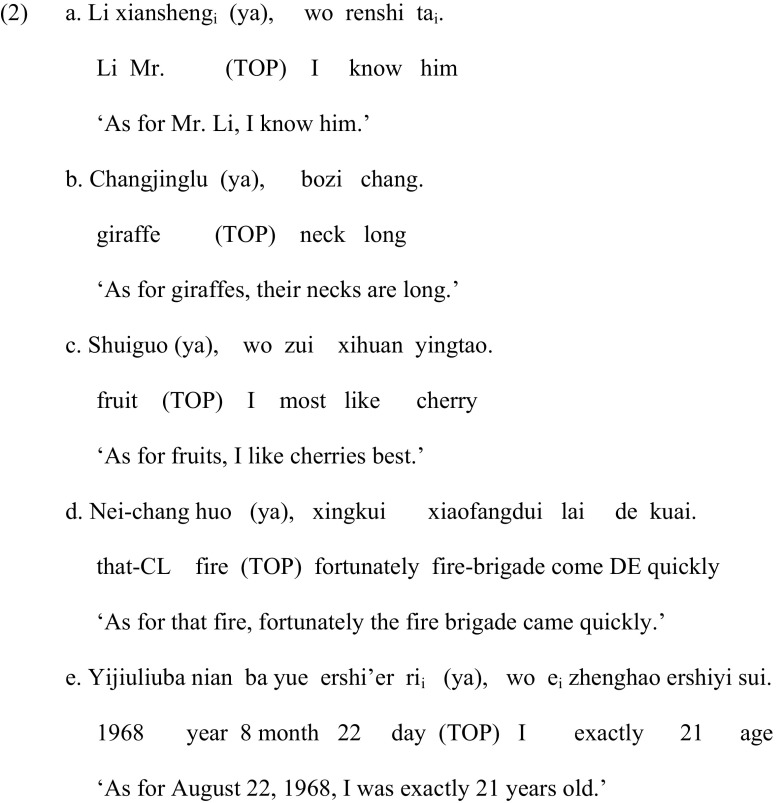


First, contrary to (1) in which the topic *Li xiansheng* ‘Mr. Li’ is related to an empty element in the comment clause, the topic in (2a) is resumed by an overt pronoun *ta* ‘him’ in the comment clause. Second, double noun constructions (‘double-subject sentences’ in Li and Thompson 1981) are also possible: the two initial NPs *changjinglu* ‘giraffe’ and *bozi* ‘neck’ in (2b) are involved in a part-whole relation, or the first NP *shuiguo* ‘fruit’ and the last NP *yingtao* ‘cherry’ in (2c) have an inclusive relation. Third, the comment as a whole may be a predicate related to the topic as in (2d), without any indication of a gap in the comment. Fourth, the topic can also be an adverbial phrase as in (2e), where the adverbial *yijiuliuba nian ba yue ershi’er ri* ‘(on) August 22, 1968’ is derived from the position after the subject *wo* ‘I’ as argued by Xu and Langendoen (1985).

Two contrasting analyses of Chinese topic structures have been proposed in the literature (see Huang et al. 2009 for complete references). According to the first family of accounts, topic structures in Chinese do not involve movement; topics are generated in their surface position. A configuration of topic structures is given in (3), adapted from Xu (2000: 29). A Topic Phrase (TopP) is the maximal projection of the Topic head, as the topic occurs in the specifier position, followed by a functional category Top and the complement of Top, i.e., IP.
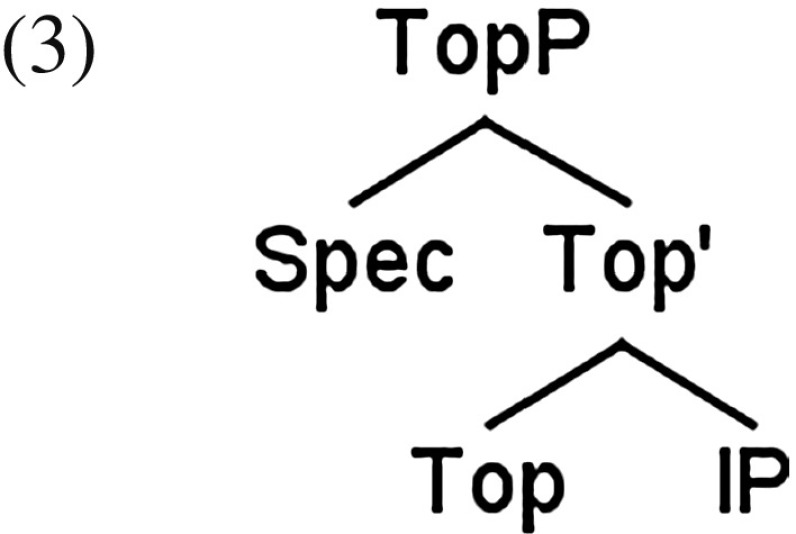


An important piece of evidence for the non-movement account comes from the ‘gapless’ topic structures exemplified in (2b–d). There is no gap in any of these sentences. An ‘aboutness’ relation between the comment clause and the topic, rather than a gap-antecedent relation, exists. These ‘gapless’ sentences have been noted by a number of linguists (Chao 1968; Li and Thompson 1976, 1981; Xu and Langendoen 1985; Xu 2000; amongst others) who further claim that all topic structures (including the sentences in (1)) are base-generated and do not involve movement.

According to the second family of accounts, not all topic structures are derived in the same manner (Huang 1982; Huang et al. 2009; Li 2000; Paul 2005; amongst others). Some topics are base-generated according to an ‘aboutness’ relation, such as the ‘gapless’ sentences in (2b–d). Other topics are associated to gaps in the comment clause, and are derived by movement. Huang et al. (2009) refer to (1) as a ‘gapped topic sentence’, contrary to ‘gapless topic sentences’ like (2b–d). Specifically, in (1) the object of the sentence *Li xiansheng* ‘Mr. Li’ has moved from the object position, leaving a gap there, and reaches the sentence-initial position. Thus, these authors assume that a gap exists in gapped topic structure and has an A’-antecedent. Evidence for the movement account comes from the displacement of part of an idiom chunk and the possibility of reconstruction (Li 2000). If topic structures are derived by movement, it is expected that they are sensitive to island conditions such as the Complex NP constraint. Related to that, let us consider (4) (example from Huang et al. 2009: 210).

**Table:** 

(4)	*Li	Si_i_,	wo	hen	xihuan	[[[t_i_	chang	ge]	de]	shengyin].
	Li	Si	I	very	like		sing	song	DE	voice
	‘*As for Li Si_i_, I like the voice with which t_i_ sings.’									

Specifically, in (4), the topic, *Li Si* ‘Li Si’, is extracted from inside a complex NP. If a resumptive pronoun is inserted in the gap position, the sentence becomes acceptable. This indicates that the ungrammaticality of (4) is not due to semantic or pragmatic anomaly, but because the topic element cannot be extracted from the complex NP island (Huang et al. 2009: 208). However, what is intriguing is the sentence (5) in which the topicalization appears grammatical notwithstanding a violation of Complex NP constraint.

**Table:** 

(5)	Li	Si_i_,	[[[t_i_	chang	ge]	de]	shengyin]	hen	haoting.
	Li	Si		sing	song	DE	voice	very	nice
	‘As for Li Si_i_, the voice with which [t_i_] sings is very nice.’								

How can one account for the ungrammaticality of (4) and the grammaticality of (5)? Huang (1984, 1989) proposed that it relates to the availability of an empty pronoun *pro* in Chinese, limited to the subject position of finite clauses in Chinese. Consider (6) (examples from Huang 1989: 187).

**Table:** 

(6)	a.	Zhang	San	shuo	[e	hen	xihuan	Li	Si].
		Zhang	San	say		very	like	Li	Si
		Interpretation 1: ‘Zhang San_i_ said that (he_i_) liked Li Si.’							
		Interpretation 2: ‘Zhang San said that (John) liked Li Si.’							
	b.	Zhang	San	shuo	[Li	Si	hen	xihuan	e].
		Zhang	San	say	Li	Si	very	like	
		‘Zhang San said that Li Si liked (John).’							
	c.	[OP_i_	[Zhang	San	shuo	[Li	Si hen	xihuan	e_i_]]].

In (6a), the null subject may refer to the matrix subject *Zhang San* (intra-sentential interpretation) or to a previously mentioned discourse topic such as *John* (extra-sentential interpretation). In (6b), the null object can only refer to a previously introduced discourse topic (i.e., only the extra-sentential interpretation is legitimate). Huang claimed that the null subject in (6a) is a null pronominal, since an overt pronoun such as *ta* ‘him’ in the same position functions in the same way, while the null object in (6b) cannot be a null pronominal, because the overt pronoun in the same position can either refer to the matrix subject *Zhang San* or to a discourse topic. Accordingly, the null object is better analyzed as a variable, which is A’-bound by an empty operator and cannot be A-bound by the matrix subject (see 6c). The comparison between (6a) and (6b) led Huang to conclude that there is a subject/object asymmetry with respect to empty categories in Chinese (for more examples, see Huang 1984, 1989). He further suggested that empty pronouns are governed by a Generalized Control Rule (GCR): *Coindex an empty pronominal with the closest nominal element* (Huang 1984: 552).

Let us go back to (4) and (5). In both examples, as Huang et al. (2009) noted, the empty category cannot be a trace of movement because that would lead to a violation of the Subjacency Condition. Alternatively, we could assume that the empty category is a *pro*, which allows base-generation. In (4), the closest antecedent of the empty category is the subject of the comment clause, *wo* ‘I’, not the topic *Li Si* ‘Li Si’. Accordingly, the sentence receives the reading ‘As for Li Si, I like my own voice of singing’ which is not comprehensible, as the topic is not related to the comment. Thus, either with a trace or with a *pro*, the sentence (4) is ill-formed. In turn, consider (5), in which the empty category is coindexed with the closest compatible antecedent and turns out to be coreferential with the topic *Li Si* ‘Li Si’. Thus, the linking between the *pro* and the topic is created without violating any principle of grammar and the sentence (5) is grammatical.

However, the subject/object asymmetry is questionable. One classical example is given in (7) (from Xu 1986: 78).

**Table:** 

(7)	Xiaotou	yiwei	[meiren	kanjian	e].
	thief	think	nobody		see
	Interpretation 1: ‘The thief_i_ thought nobody saw (him_i_).’				
	Interpretation 2: ‘The thief thought nobody saw (John).’				

The null object is naturally interpreted as the matrix subject *xiaotou* ‘thief’, but within an appropriate context it can refer to the discourse topic *John*. It seems that the syntactic restrictions can be overridden by semantic and pragmatic plausibility. Thus, the distribution of the empty pronoun is not limited to the subject position of finite clauses as Huang (1989) claimed, but may also include the object position of finite clauses (for alternative approaches see Xu 1986 and Li 2014).

In sum, although the evidence from the linguistic literature is controversial, the hypothesis that movement is involved at least in some topicalization structures cannot be discarded.

### Previous Studies on the Acquisition of Chinese Topic Structures

Previous studies on how children acquire topicalizations reveal the existence of cross-linguistic variation: in some languages, topicalization seems to be difficult for children at age four (e.g., in Japanese, see Sano 2005), while in others it is acquired by 4 years of age (e.g., in German, see Spinner and Grinstead 2006). In the interest of space, we only review some previous studies on Chinese.

In Chinese, the existence of ‘gapless topic sentences’ and ‘gapped topic sentences’, as discussed above, partly obscures the possibility of distinguishing between subject and topic. Consider (8).

**Table:** 

(8)	a.	Li	xiansheng_i_	(ya),	wo	renshi	e_i_.	(OSV topicalization)
		Li	Mr.	(TOP)	I	know		
		‘As for Mr. Li, I know (him).’						
	b.	Li	xiansheng	(ya),	renshi	wo.		
		Li	Mr.	(TOP)	know	me		
		Interpretation 1: ‘As for Mr. Li, (he) knows me.’						(SVO topicalization)
		Interpretation 2: ‘Mr. Li knows me.’						(canonical SVO)

In the literature, not only (8a) but also (8b) have been regarded as topic structures (Li and Thompson 1976, 1981; Xu and Liu 2007). In object topicalization sentences (with the OSV order), the topic and the subject are distinct, whereas in the SVO sentences the topic and the subject share the same position, at least superficially. In (8a), we can identify the topic and the subject regardless of the presence of the topic marker (i.e. *ya*). The topic is *Li xiansheng* ‘Mr. Li’ and the subject is *wo* ‘I’. The topic occurs in sentence-initial position and is related to an empty element in the comment clause. However, in (8b), the topic and the subject are identical. With a topic marker such as *ya*, the sentence receives the topic interpretation (Interpretation 1); without the topic marker, the sentence can be regarded as a canonical SVO sentence (Interpretation 2) or as a topicalization sentence with the subject being the topic given the proper context (Interpretation 1). Consider (9).

**Table:** 

(9)	Speaker A:	Li	xiansheng	shi	women	xinlai	de	jingli.		
		Mr.	Li	is	we	new-coming	DE	manager		
		‘Mr. Li is our new-coming manager.’								
	Speaker B:	*Li*	*xiansheng*	*(ya),*	*renshi*	*wo*.	Women	shi	daxue	tongxue.
		Mr.	Li	(TOP)	know	me	we	are	university	classmate
		‘As for Mr. Li, (he) knows me. We were classmates at university.’								

In the sentence by speaker B, *Li xiansheng* ‘Mr. Li’ has been mentioned in the previous discourse (Speaker A) and is being discussed again (Speaker B). It can be followed by an optional topic marker such as *ya*, and the remaining clause *renshi wo* ‘know me’ is about this specific person. Given that the phrase satisfies the conditions for being the topic, we consider the sentence as a topic structure.

To examine whether Chinese children distinguish topic and subject at the early stages, Chien and Lust (1985) tested 95 Mandarin-speaking children aged from 2.6 to 5.0 by using an elicited imitation task. They asked children to imitate coordinate sentences like (10a) and control-sentences like (10b).

**Table:** 

(10)	a.	*Baobao*,	jiao	hen	xiao;	*baobao*	ye	hen	ke’ai.
		baby	foot	very	small	baby	also	very	cute
		‘As for the baby, the feet are small; as for the baby, (he) is also very cute.’							
	b.	*Xiaohua*,	jiejie		xihuan	*Xiaohua*	dai	maozi.	
		Xiaohua	older	sister	like	Xiaohua	wear	hat	
		‘As for Xiaohua, (his) older sister likes Xiaohua to wear a hat.’							

The results of the experiment showed that children dropped the second NP (i.e. *baobao* ‘baby’) much more frequently than the first NP (i.e. *baobao* ‘baby’) in the coordinate construction (25.74 vs. 2.38%). They were more likely to omit the first NP (i.e. *Xiaohua* ‘Xiaohua’) than the second NP (i.e. *Xiaohua* ‘Xiaohua’) in the control-construction (48.59 vs. 3.36%). Based on the fact that children were able to reduce redundant topics, Chien and Lust suggested that young children were sensitive to the topic-subject distinction to some degree. However, the findings were confounded by problems with the experimental materials used, e.g., in (10b) instead of treating the first *Xiaohua* ‘Xiaohua’ as the topic, *Xiaohua jiejie* can be interpreted as the subject of (10b) with the genitive maker *de* omitted (i.e. *Xiaohua de jiejie* ‘Xiaohua’s older sister’).

A few studies have looked at children’s production of topic structures. Erbaugh (1982, 1992) conducted a longitudinal study with 4 Mandarin-speaking children aged 1:10 to 3:10 and found that topicalization was not frequent in the early stage of development and in many observed sentences the comment was truncated or anaphorically unclear. Hendriks (2000) examined narratives produced by 5-, 7-, and 10-year-old Chinese children and adults (10 per group). The results showed that most topics were subjects, which were highly active in discourse, and no object topicalization was found. The author also examined the occurrence of topic markers *ne*, *ba*, and *a*, and found that they were not frequent, with only 31 such utterances produced by 5-year-olds, 3 utterances produced by 7- and 10-year-old children and 29 utterances by adults.

Chen (2009) analyzed the speech of 44 Mandarin-speaking children from the CHILDES database (MacWhinney 2000). She divided them into four age groups: the 2:2 age group (N = 10), the 2:8 age group (N = 10), the 4:0 age group (N = 12) and the 6:0 group (N = 12). Contrary to Hendriks (2000), the author did not code subject topicalization, and, following the idea that overt topic markers are optional, she did not examine whether topic structures had an overt topic marker. The results showed that children began to produce object topicalization sentences like (1) as early as age 2:2, but only 5 sentences (1.3%, out of 387 utterances) were found. The 2:8 age group produced only 8 such sentences (3.5%, out of 227 utterances), including 7 object topicalization sentences like (1) and 1 adverbial topic like (2e). In the age group 4:0 and 6:0, more topic sentences were found, 34 sentences (3.9%, out of 875 utterances) and 35 sentences (3.5%, out of 1009 utterances) respectively. Of them, only 5 sentences were object topicalizations in each age group. The author also examined a small sample of adult data, represented by four TV talk shows. The percentage of topic sentences produced by adults ranged from 2.02 to 5.17%. Thus, although topic structures are produced from a very early age, they are not abundant in the spontaneous speech of Chinese children and adults.

To sum up, previous studies have shown that topic structures are produced by Chinese children from their first multiword combinations, but they are rare in spontaneous speech. However, it is difficult to draw conclusions on the basis of the current literature. The first problem is the treatment of topic markers. As pointed out above, the use of overt topic markers may be optional, but which topic marker is used is often related to specific contexts. It is crucial to disentangle the acquisition of topic structures with an overt topic marker from those without an overt topic marker. The second problem has to do with the ages of the children examined. Object topicalization was produced by children at age 2:2 in Chen (2009), but not by older children in Hendriks (2000). The contrasting results may be due to the different methods to collect the data, so it is impossible to draw meaningful generalizations. The third problem is the lack of data on comprehension so far. Previous studies have examined adult processing of topicalization, but the results are not clear either (Cai and Dong 2010; Huang and Kaiser 2008; Yang and Liu 2014). These problems, together with the need to find evidence for or against the movement analysis of topicalization, motivate our study on the acquisition pattern of topicalization with and without an overt topic marker and the acquisition pattern of subject/object topicalization.

### Two Hypotheses

In this section, first we introduce the Relativized Minimality account (RM; Rizzi 1990, 2004; Starke 2001); then, we consider the movement and non-movement analyses of topicalization and their predictions for acquisition within the RM framework.

According to the RM principle, a local relation between X and Y cannot be established if an intervening element Z, having the same feature specification as X, acts as a potential candidate for the same relation (Rizzi 1990).

**Table:** 

(11)	X	_…_	Z	_…_	Y

Based on RM expressed in terms of features (Rizzi 2004; Starke 2001), three different types of relation between the target X and the intervener Z are given: identity, inclusion and disjunction (Friedmann et al. 2009; Belletti and Rizzi 2013). In the configurations in (12), A and B stand for abstract morphosyntactic features. First of all, when the intervener’s and the target’s featural specifications are identical as in (12a), RM rules out the sentence (e.g. *How do you wonder who could solve this problem?*). Second, when the intervener’s featural specification is included in the target’s featural specification as in (12b), the structure (e.g. *Which problem do you wonder how to solve?*) is ruled in by the RM principle (although some degradation is observed). Finally, when the featural specification of the intervener and that of the target are disjoint as in (12c), the structure (e.g. *What do you think John solved?*) is ruled in by RM.

**Table:** 

(12)		X		Z		Y	
	a.	+A	……	+A	……	<+A>	(identity)
	b.	+A, +B	……	+A	……	<+A, +B>	(inclusion)
	c.	+A	……	+B	……	<+A>	(disjunction)

RM holds similarly in adult and child grammar, so identity of feature specification as in (12a) leads to ungrammaticality and disjunction of feature specification as in (12c) results in grammaticality, but indeed some differences between the adult and the child system arise concerning (12b). According to Friedmann et al. (2009), the adult system permits an A’-moved element to cross over an intervener as long as the intervener has a distinct feature specification, so both the configurations (12b) and (12c) lead to a grammatical output in the adult system. In contrast, the child system is argued to allow movement only when the specification of the intervener is disjoint from that of the A’-moved element as in (12c), but does not admit (12b) because of the difficulty of computing subset-superset relations of features. To consider the target and the intervener distinct as in (12b), one has to compute the subset relation, but (young) children’s limited computational resources sometimes hinder them from making that complex computation. This line of explanation has captured locality effects linked to intervention in relative clauses, topicalization, control structures, passives and *wh*-questions by agrammatic Broca’s aphasics (Garraffa and Grillo 2008; Grillo 2008, 2009) and children’s difficulty in acquiring object relative clauses and object *which*-questions (e.g., Belletti and Rizzi 2013; Friedmann et al. 2009; for alternative approaches, see Goodluck 2010; Bentea and Durrleman 2018). Within the RM framework, a more detailed definition of the feature specification of the intervener which modulates intervention effects in A’-dependencies has been further proposed to account for children’s performance (see Adani et al. 2010, for evidence on how number and gender features affect Italian children’s performance; Belletti et al. 2012, Guasti et al. 2012, for elaborated analyses on how the differential status of morphosyntactic features in languages affects the computation of intervention; Costa et al. 2012, for evidence that intervention effects can emerge even in the absence of lexical restrictions; Hu et al. 2016a, b for extending the RM approach to explain children’s acquisition of Mandarin relative clauses, a structure which shares many properties with topic structures).

In the context of these findings, now we focus our attention on object topicalization (with OSV order) as in (13a) and subject topicalization (with SVO order) as (13b). As discussed above, with the appropriate context, the two structures constitute a minimal pair of topicalization sentences.

**Table:** 

(13)	a.	Zhe-ge	haizi_i_	(ya),	waipo	zai	hua	e_i_.
		this-CL	child	(TOP)	grandma	PROG	draw	
		‘As for this child, the grandma is drawing (him).’						
	b.	Zhe-ge	haizi_i_	(ya),	e_i_	zai	hua	waipo.
		this-CL	child	(TOP)		PROG	draw	grandma
		‘As for this child, (he) is drawing the grandma.’						

If topicalization involves A’-movement, RM applies to the structure, and in that case the sentences in (13) have the structure in (14) in which ‘D NP’ stands for a nominal expression. The first ‘D NP’ is the topic phrase, which is attracted to the left peripheral position by the bundle of features [+ TOP, +NP], where ‘TOP’ designates the topic feature expressed overtly or covertly by the topic marker, and ‘NP’ designates the nominal feature that the topic also bears. We adopt the feature [+ NP] as in the RM framework, where it is understood as a categorial feature, an assumption that may be problematic. Therefore, we suggest that this feature might be a referential feature.

**Table:** 

(14)	a.	D NP	……	D NP	……	<D NP>	(OSV topicalization)
		[+TOP, +NP]		[+NP]			
	b.	D NP	……	<D NP>	……	D NP	(SVO topicalization)
		[+TOP, +NP]				[+NP]	

In (14a), the subject of the comment also bears a [+ NP] feature and intervenes between the topic and its copy. According to the RM approach, in the configuration (14a), a RM violation may be detected by children because they may not consider the featural specification of the topic and that of the subject distinct. In order to consider them distinct, a subset relation has to be computed, but (young) children are assumed to have limited computational resources which would prevent them from making the computation. In contrast, no problem arises in SVO topicalization sentences as illustrated in (14b), because there is no intervener between the topic and its copy. In summary, we expect children to display difficulties in comprehending OSV topicalization sentences compared with SVO topicalization sentences, if topicalized structures are derived by A’-movement.

As we mentioned before, a non-movement analysis is also possible for topicalization sentences. The topic is merged in the topic position in the left periphery of the clause and it entertains a binding relation with a base-generated *pro* as in (15) (an analysis that is, however, at odds with Huang 1984, 1989).

**Table:** 

(15)	a.	Zhe-ge	haizi_i_	(ya),	waipo	zai	hua	pro_i_.
		this-CL	child	(TOP)	grandma	PROG	draw	
		‘As for this child, the grandma is drawing (him).’						
	b.	Zhe-ge	haizi_i_	(ya),	pro_i_	zai	hua	waipo.
		this-CL	child	(TOP)		PROG	draw	grandma
		‘As for this child, (he) is drawing the grandma.’						

This *pro* is coindexed with the nominal element like an overt pronoun. In fact, previous studies have showed that Chinese children treated *pro* and an overt pronoun quite similarly with regard to coreference interpretations (Lust et al. 1996). Under this analysis, the relation between the topic and *pro* is an anaphoric relation, not one derived from movement. Since movement does not occur, RM does not apply. Therefore, we do not expect any difference in the comprehension of OSV topicalization sentences and SVO topicalization sentences, if the non-movement analysis is adopted.

Summarizing, two contrasting hypotheses are proposed below.

**Table:** 

(16)	**Hypothesis 1** Under the movement analysis, topicalization involves A’-movement and the usual locality principles apply, in particular, RM does. Thus, OSV topicalization sentences are predicted to be harder than SVO topicalization sentences in acquisition, under the assumptions of RM.
	**Hypothesis 2** Under the non-movement analysis, an anaphoric relation holds between the topic and the empty category *pro*. RM does not apply in this case as there is no movement. Thus, no asymmetry between OSV topicalization sentences and SVO topicalization sentences is expected in acquisition.

## Experiment 1

In our study, we aimed at evaluating the two hypotheses above. We did this through two experiments on the acquisition of Chinese subject and object topicalizations, one with the presence of an overt topic marker (Experiment 1) and another in the absence of an overt topic marker (Experiment 2). In Experiment 1, an overt topic marker *ya* was used because it is almost equal to a simple pause (Chu 2003) and in Experiment 2 no overt marker was used. To evaluate whether sentences with overt topic markers and covert topic markers are natural, a list of 4 sentences as in (15) were prepared and 12 adults were asked to emit a judgement on a scale rating from 0 (*not at all natural*) to 10 (*extremely natural*). The results showed that the mean of sentences with an overt topic marker were numerically lower than those with covert topic marker (M = 6.17, SD = 2.66; M = 7.12, SD = 3.11, respectively), but no significant difference between them was found (t (23) = 1.66, *p* > .05). By carrying out two experiments, we can make a clear-cut distinction between overt topic markers and covert topic markers.

### Participants

Sixty-six Mandarin-speaking children aged from 3:0 to 6:11 participated in this experiment. They were divided into four age groups: the 3-year-old group (N = 16, aged 3:0–3:11, M = 3:8, SD = .25, 7 males), the 4-year-old group (N = 16, aged 4:0–4:11, M = 4:5, SD = .23, 8 males), the 5-year-old group (N = 18, aged 5:1–5:11, M = 5:6, SD = .26, 9 males) and the 6-year-old group (N = 16, aged 6:2–6:11, M = 6:5, SD = .20, 8 males). They lived in Zhejiang, China and were developing normally. An additional adult group (N = 10, aged 24:3–29:10, M = 26:8, SD = 1.54, 5 males) served as control.

### Materials and Design

The stimuli consisted of 8 OSV topicalization sentences like (17a) and 8 SVO topicalization sentences like (17b). An overt topic marker, *ya*, was used. We used 8 transitive verbs: *bang* ‘help’, *da* ‘hit’, *gai* ‘cover’, *hua* ‘draw’, *kan* ‘look at’, *tui* ‘push’, *yao* ‘bite’ and *zhui* ‘chase’.

**Table:** 

(17)	a.	Zhe-zhi	qingwa	ya,	laoshu	zai	da.
		this-CL	frog	TOP	mouse	PROG	hit
		‘As for this frog, the mouse is hitting (it).’					
	b.	Zhe-zhi	xiaogou	ya,	zai	da	xiaomao.
		this-CL	dog	TOP	PROG	hit	cat
		‘As for this dog, (it) is hitting the cat.’					

All of the experimental sentences were semantically reversible, and the noun phrases were animate. In addition, there were 8 filler sentences including verbs that were not semantically reversible (e.g., *Zhe*-*ge nanhai, zai kan shu* ‘As for this boy, (he) is reading books’), and that did not include an overt topic marker. See the complete list in the Appendix.

**Fig. 1:**
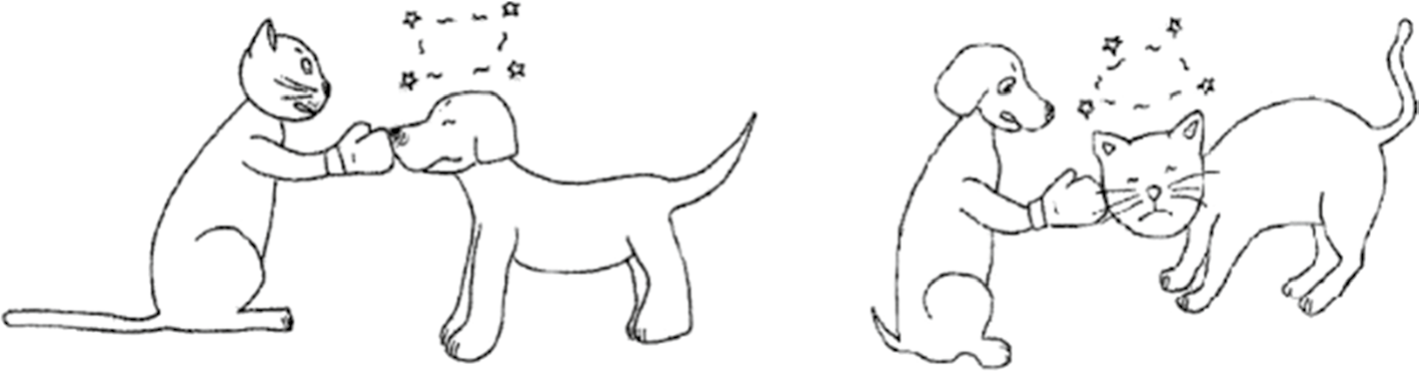
A set of experimental pictures used in the present study

Each experimental sentence was associated with a set of experimental pictures as exemplified in Fig. 1. In particular, Fig. 1 was associated to the Chinese equivalent of a topicalization sentence, i.e., ‘As for this dog, (it) is hitting the cat’. In total, there were 24 sets of experimental pictures. As all the pictures had been previously used in the study of Chinese children’s acquisition (Hu et al. 2016b), it seems safe to assume that the pictures themselves cannot be the source of miscomprehension on the part of children. The stimuli and the fillers were presented to each participant in pseudo-random order.

To make the use of topicalization felicitous, we created an appropriate context. The experimenter asked participants to describe the picture first, e.g., to name the characters, *xiaogou* ‘dog’ and *xiaomao* ‘cat’ in Fig. 1, or to calculate the numbers of the characters, *liang*-*zhi xiaogou* ‘two dogs’ and *liang*-*zhi xiaomao* ‘two cats’ in Fig. 1. Then, the experimenter told children: *Wo zai kan yi*-*zhi xiaogou* ‘I am looking at a dog’. By doing this, we provided a context for topicalization. Next, the experimenter presented the target sentences orally, e.g., *Zhe*-*zhi xiaogou ya, zai da xiaomao.* ‘As for this dog, (it) is hitting the cat’, and the participant was asked to point to the picture matching the sentence.

Participants were tested individually. Each participant was asked to look at the experimental pictures on an iPad screen. After providing an appropriate context for topicalization, the experimenter presented the target sentence and the participant pointed to a picture (out of two). Two practice items were presented to ensure that participants understood the task.

### Results

The experiment yielded a total of 1056 responses from children and 160 from adults (excluding responses to fillers, because all the fillers were answered correctly). Half of the responses corresponded to OSV topicalizations and the other half to SVO topicalizations. Table 1 shows the responses in each condition across age groups. The percentages, the raw scores, the means and the standard deviation were calculated by group and by sentence type. The descriptive analysis showed that children’s comprehension of SVO topicalization was more accurate than OSV topicalization, and the accuracy of both structures improved from three to 6 years of age. Adults performed at ceiling, with 100% correct responses in both sentence type conditions.

**Table 1:** Percentages (%), raw scores (N), means (M) and standard deviation (SD) of correct responses in each age group

Groups	OSV topicalization				SVO topicalization			
	%	N	M	SD	%	N	M	SD
3 y.o.	55	70/128	5.69	1.70	71	91/128	4.38	1.02
4 y.o.	63	81/128	6.25	1.39	78	100/128	5.06	1.65
5 y.o.	89	128/144	7.44	1.54	93	134/144	7.11	1.78
6 y.o.	96	123/128	7.81	0.75	98	125/128	7.69	1.25
Adult	100	80/80	8.00	0.00	100	80/80	8.00	0.00

We analyzed the data using the lme4 package in the R environment (Bates et al. 2013; R Core Team 2015). We fit the data with linear mixed-effects models, including sentence type (i.e., OSV vs. SVO) and age (i.e., 3-, 4-, 5- vs. 6-year-olds) as fixed factors, and subjects and items as random-effect factors. The reference categories were the SVO sentences for the sentence type factor and the 3-year-olds for the age factor.

First of all, sentence type yielded a significant effect (χ^2^ (1) = 7.03, *p* < .01; Wald *Z *= − 2.97, *p* < .01). We further compared the two structures in each age group, and found that only the 3-year-old group and the 4-year-old group performed significantly better in SVO topicalization sentences than in OSV topicalization sentences (χ^2^ (1) = 4.54, *p* < .05; Wald Z = − 2.27, *p* < .05, and χ^2^ (1) = 4.38, *p* < .05; Wald Z = − 2.25, *p* < .05, respectively), suggesting that there is a subject/object asymmetry on the comprehension of Chinese topicalizations in 3- and 4-year-old children.

Second, age yielded a significant effect (χ^2^ (3) = 51.51, *p* < .001). By changing the reference categories, we compared each age group with the other age groups. Table 2 reports the output of the analysis. There was no significant improvement in accurate responses from age three to age four. Crucially, a robust improvement occurred at 5 years of age, namely, the performance of 3- and 4-year-olds significantly differed from that of 5-year-olds. There was no significant difference between age five and age six.

**Table 2:** Summary of the age factor in the mixed-effects model (N = 1056, SD of subjects = 1.42, SD of items = 0.58, log-likelihood = − 404.55) in the comprehension of the two structures

Age groups	Estimate	SE	Wald *Z*	*p*
3 y.o. versus 4 y.o.	0.53	.54	0.99	= .33
3 y.o. versus 5 y.o.	3.03	.62	4.87	< .001
3 y.o. versus 6 y.o.	4.57	.89	5.13	< .001
4 y.o. versus 5 y.o.	2.48	.63	3.97	< .001
4 y.o. versus 6 y.o.	4.04	.90	4.51	< .001
5 y.o. versus 6 y.o.	1.55	.94	1.64	= .10

To summarize, children, specifically at age three and four, comprehended SVO topicalization sentences much better than OSV topicalization sentences. Improvement in both structures was observed, and essentially a robust improvement occurred at 5 years of age.

## Experiment 2

The second experiment aimed at verifying whether the same subject/object asymmetry holds true when there is no overt topic marker in topicalization sentences.

### Participants

A different group of participants were recruited in Experiment 2, including 80 Mandarin-speaking children aged from 3:0 to 6:11. They were divided into four age groups: the 3-year-old group (N = 20, aged 3:0–3:9, M = 3:3, SD = .26, 10 males), the 4-year-old group (N = 20, aged 4:0–4:11, M = 4:4, SD = .31, 8 males), the 5-year-old group (N = 20, aged 5:0–5:11, M = 5:5, SD = .32, 9 males) and the 6-year-old group (N = 20, aged 6:0–6:11, M = 6:6, SD = .29, 11 males). As in Experiment 1, all the children lived in Zhejiang, China and were developing normally. An additional adult group (N = 10, aged 25:7–28:11, M = 26:7, SD = 1.11, 5 males) served as control.

### Materials and Design

The materials used in Experiment 2 were identical to those used in Experiment 1 except that an overt topic marker was not used in the experimental sentences, as illustrated in (18a) for OSV topicalization sentences and in (18b) for SVO topicalization sentences.

**Table:** 

(18)	a.	Zhe-zhi	qingwa,	laoshu	zai	da.
		this-CL	frog	mouse	PROG	hit
		‘As for this frog, the mouse is hitting (it).’				
	b.	Zhe-zhi	xiaogou,	zai	da	xiaomao.
		this-CL	dog	PROG	it	cat
		‘As for this dog, (it) is hitting the cat.’				

To iterate, we aimed at making a clear-cut distinction between overt topic markers and covert topic markers, so we did not use the intonational break in Experiment 2. As in Experiment 1, we provided an appropriate context to make the use of topicalization felicitous. Given that the initial phrase of the sentence is a topic, both structures regardless of word order (OSV vs. SVO) are topicalizations.

### Results

The experiment yielded a total of 1280 responses from children and 160 from adults. Half corresponded to the comprehension of OSV topicalizations and the other half to SVO topicalizations. As in Experiment 1, we excluded the analysis of filler sentences, as all were answered correctly. Table 3 shows the percentages, the raw scores, the means and the standard deviation in each condition across age groups. Interestingly, both SVO and OSV topicalization sentences were comprehended well. As in Experiment 1, the accuracy of the two structures improved from 3 to 6 years of age, and adults comprehended all the sentences correctly 100% of the time.

**Table 3:** Percentages (%), raw scores (N), means (M) and standard deviation (SD) of correct responses in each age group

Groups	OSV topicalization				SVO topicalization			
	%	N	M	SD	%	N	M	SD
3 y.o.	76	121/160	6.05	1.50	88	141/160	7.05	0.89
4 y.o.	84	134/160	6.70	1.26	89	142/160	7.10	1.02
5 y.o.	93	149/160	7.45	0.10	96	154/160	7.70	0.57
6 y.o.	98	156/160	7.80	0.52	100	160/160	8.00	0.00
Adult	100	80/80	8.00	0.00	100	80/80	8.00	0.00

First of all, the sentence type factor did not predict the comprehension ability (χ^2^ (1) = 2.71, *p* = .10; Wald *Z *= − 1.70, *p* = .09). Neither within the 3-year-old group, nor within the 4-year-old group did we find any significant difference between the two sentence types (χ^2^ (1) = 2.11, *p* > .05; Wald Z = − 1.55, *p* > .05, and χ^2^ (1) = 1.03, *p* > .05; Wald Z = − 1.02, *p* > .05, respectively). The results indicate that comprehension of topicalization sentences with the OSV order and those with the SVO order did not differ.

Second, age yielded a significant effect (χ^2^ (3) = 38.03, *p* < .001). As is evident in the output of the analysis (Table 4), there was no significant improvement in accurate responses from age three to age four. Crucially, we observed a robust improvement at 5 years of age, showing that the performance of 5-year-olds significantly differed from those of 3- and 4-year-olds. There was a significant difference also between age five and age six.

**Table 4:** Summary of the age factor in the mixed-effects model (N = 1280, SD of subjects = 0.95, SD of items = 1.04, log-likelihood = − 335.56) in the comprehension of the two structures

Age groups	Estimate	SE	Wald *Z*	*p*
3 y.o. versus 4 y.o.	0.39	.39	1.02	= .31
3 y.o. versus 5 y.o.	1.66	.45	3.73	< .001
3 y.o. versus 6 y.o.	3.22	.67	4.80	< .001
4 y.o. versus 5 y.o.	1.26	.45	2.80	< .01
4 y.o. versus 6 y.o.	2.82	.67	4.19	< .001
5 y.o. versus 6 y.o.	1.56	.71	2.20	< .05

We further compared the results of the two experiments (see Tables 1, 3), and observed that the accuracy rates in Experiment 1 were lower than those in Experiment 2. This observation is supported by the statistical analysis. Topic marker (with an overt topic marker in Experiment 1 versus without an overt topic marker in Experiment 2) was entered into a factorial model, and significantly contributed to the model fit (χ^2^ (1) = 6.05, *p* < .05; Wald Z = 2.57, *p* < .05). We contrasted the difference for each age group, and found that the significant difference was evident in the 3-year-old group (χ^2^ (1) = 14.57, *p* < .001; Wald Z = 4.16, *p* < .001) and in the 4-year-old group (χ^2^ (1) = 9.29, *p* < .01; Wald Z = 3.29, *p* < .01).

To summarize, when an overt topic marker was not used in topicalizations, children comprehended OSV and SVO topicalization sentences equally well from age three and there was no significant difference between the comprehension of the two structures at any age. Improvement in both structures was observed, and a robust improvement was found at 5 years of age. Besides, as we compared the results of two experiments, we found that the 3- and 4-year-old children’s comprehension of Chinese topicalization with an overt topic marker (Experiment 1) was significantly worse than that of Chinese topicalization without an overt topic marker (Experiment 2).

## General Discussion

As it is evident in Experiment 1, children comprehended significantly better subject topicalizations (with SVO order) than object topicalizations (with OSV order) when the topic marker is overt in the structure. Specifically, there is a subject/object asymmetry on the acquisition of Chinese topicalization with an overt topic marker at 3 and 4 years of age (i.e., at age three, 71 vs. 55%; at age four, 78 vs. 63%). This contrasts with children’s performance on topicalizations without an overt topic marker in Experiment 2: although the accurate rates of subject topicalizations were numerically higher than object topicalizations (e.g. at age three, 88 vs. 76%; at age four, 89 vs. 84%), no difference between the two structures reached significance in any age group. Moreover, we noticed that the accuracy rate in Experiment 2 was much higher than in Experiment 1, specifically at age three and four. These findings indicate that children treat the structure with an overt topic marker and that without an overt topic maker differently in the early stage of development, and certainly suggest that topic markers such as *ya* are not just optional elements.

Two analyses are offered in the literature on topic structures: the movement analysis and the base-generation analysis. They make different predictions for acquisition when combined with the RM approach, as we discussed before. According to the RM principle, a local relation between X and Y cannot hold if Z has the same feature specification as X and acts as a possible candidate for the same relation, as in (19a). If topicalization involves A’-movement, RM applies as in (19b). Since we assume that (young) children have trouble computing the subset relation, they may not consider the topic and the subject distinct and may consider (19b) a RM violation. In this case, we predict that object topicalization would be harder than subject topicalization. On the other hand, if topicalization does not involve A’-movement and the topic is base-generated in the left periphery, RM does not apply and no asymmetry between subject and object topicalizations is expected.

**Table:** 

(19)	a.	X	……	Z	……	Y
	b.	D NP	……	D NP	……	<D NP>
	[+TOP, +NP] [+NP]					

The observed subject/object asymmetry in Experiment 1 is consistent with the predictions of the movement analysis, as children at 3 and 4 years of age did show great difficulty in object topicalization. The lack of asymmetry in Experiment 2 seems to support the base-generation analysis, as children do not seem to have trouble establishing the relation between the topic and the empty category in the comment clause, in spite of the presence of the subject. However, one may question this explanation on different grounds, which we consider below.

One may argue that the greater difficulty of object topicalization with an overt topic marker in Experiment 1 is not due to the intervener c-commanding the goal and having a subset of features of the moved element as predicted by the RM approach, but is simply due to the effects of linear intervention. Friedmann and Costa (2010) investigated coordination sentences with crossing dependencies (*The girl kissed the boy and went to the beach* in which the subject of the first conjunct is the subject of the second conjunct) and object relative clauses (*The girl that the boy kissed*). The former structure presumably involves no syntactic movement, while the latter involves A’-movement. They tested Hebrew and European Portuguese speaking children aged 3–5 and found that, in both languages, children’s difficulty in understanding the coordination sentences with crossing dependencies resembled the difficulty in object relative clauses. The authors ascribed the difficulty of the coordination sentences with crossing dependencies to the effects of linear intervention, as in this structure the intervener (*the boy)* linearly precedes the null subject of the second conjunct, without c-commanding it. Accordingly, the authors concluded that children’s difficulty with crossing dependencies is not restricted to A’-movement phenomena. In the present study, regardless of whether the topic marker is overt, in object topicalization sentences, the subject of the comment intervenes at least linearly between the topic and the object position, while in subject topicalization sentences there is no such a linear intervener. If we generalize Friedmann and Costa’s (2010) finding, we would expect object topicalization to be harder than subject topicalization, regardless of whether there is an overt topic marker or not. However, this is not confirmed by our study, as we only observed children’s difficulty in the case of object topicalization with an overt topic marker, but not in the case of object topicalization without an overt topic maker (see also Hu et al. 2016b for further discussion of linear intervention).

One may also argue that the lack of asymmetry in topicalization without an overt topic marker in Experiment 2 is due to the fact that the children might use some superficial strategy that involves no topicalization or crossing dependency. For instance, they might adopt a strategy whereby the noun phrase that immediately precedes the verb is interpreted with an agentive bias, i.e., take the pre-verbal NP as the agent. With such a strategy, they would answer correctly in both subject and object topicalizations without an overt topic marker, regardless of any RM or inclusion feature deficits. However, we think this is not the case. If children had used such a strategy, they would have comprehended object topicalization with an overt topic marker as well as object topicalization without an overt topic marker, since in both constructions the preverbal NP can be interpreted with an agent bias. Again this is inconsistent with the experimental results.

In our view the most parsimonious analysis is that topicalization with an overt topic marker and topicalization without an overt topic marker are derived in different ways in child grammar: the former through A’-movement and the latter through base-generation. This solution runs against one problem, which we discussed earlier. Extraction out of islands leads to ungrammatical sentences and topicalization out of a complex NP is ungrammatical, whether the topic marker is present or not, as is evident in (20). This means that both structures must be derived by movement.

**Table:** 

(20)	a.	*Li	Si_i_	ya,	wo	hen	xihuan	[[[t_i_	chang	ge]	de]	shengyin].
		Li	Si	TOP	I	very	like		sing	song	DE	voice
	b.	*Li	Si_i_,	wo	hen	xihuan		[[[e_i_	chang	ge]	de]	shengyin]
		Li	Si	I	very	like			sing	song	DE	voice
		‘*As for Li Si_i_, I like the voice with which t_i_ sings.’										

However, it is generally agreed that topicalization does not involve movement at least in the case of ‘gapless’ topic structures (Huang et al. 2009, but see Shi 2000 for a different view). Thus, children have evidence that in some cases topicalization cannot be derived by movement. We propose that based on this evidence children initially analyze subject and object topicalization structures without an overt topic marker as being based-generated as such. If this conjecture is correct, these children should judge (20b) acceptable, but we leave this prediction for future research. Since children analyze topic structures without an overt topic marker as being base-generated structure, it is not surprising that no subject/object asymmetry was observed in Experiment 2, in line with the prediction of the RM approach. On the other hand, this option is not available in the case of structures in which the topic marker is overt. In fact, it can be argued that the topic marker is the morphological expression of the feature that attracts the topic, via movement, to the left periphery. Hence, in this case a subject/object asymmetry is expected, as predicted by the RM approach. In the adult grammar, topicalization structures with a gap, with or without the topic marker, are derived by movement, as proven by (20). This means that children have to abandon the base-generation analysis and they do that when they acquire the syntax and semantics of the various topic markers. In other words, structures with overt topic markers are evidence that movement is required in topicalizations, where the overt topic markers attract the topic to the left periphery; in structures without the topic marker, the attractor will be an empty topic marker.

Furthermore, we suggest that children need time to acquire the syntax and semantics of topic markers, as shown by the fact that the accuracy rate of even subject topicalization with the topic marker *ya* (71%), at age three, was quite differently from that of subject topicalization without an overt topic marker (88%), and was more similar to that of object topicalization without an overt topic marker (76%). The same pattern holds for the 4-year-old group. This fact suggest that children are not ignoring the topic marker, but they do not master it fully at age three and four. Firstly, as mentioned earlier, the discourse pragmatic roles of each topic marker are different (Chu 2003, 2006; Lee 2003; Yuan 2003; Zhang and Fang 1996). Secondly, sentences with different topic markers might have different structures. For example, based on German and Italian, Frascarelli and Hinterhölzl (2007) reported that three types of topics (i.e. aboutness topic, contrastive topic and familiar topic) are distinguished phonologically and realized in different syntactic positions. The same may hold for Mandarin Chinese, something that needs to be addressed in the future.

To sum up, this study used a picture-sentence matching task with an appropriate context to investigate the comprehension of topicalization with two different orders in Chinese by testing young children. The results revealed that children displayed good comprehension of both subject and object topicalizations without a topic marker (Experiment 2), but, when the topic marker is overt, their comprehension accuracy decreased for both subject topicalizations and object topicalizations (Experiment 1). Critically, these contrasting findings indicate that topic markers such as *ya* are not just something optional, but play a critical role in child acquisition. We followed previous analyses and assumed that both structures involve movement in the adult grammar. However, children initially analyze sentences without an overt topic marker as being base-generated. They abandon this analysis in favor of the movement analysis based on the evidence provided by sentences including overt topic markers. And this happens when children master their syntax and semantics, which takes some time. The acquisition data from the current study, interpreted in the RM framework that we have assumed, point to the conclusion that topicalization in some cases is not derived by movement and children avail themselves of this option for a while.

## Appendix

The only difference between the experimental sentences of the two experiments was that an overt topic marker *ya* was used in Experiment 1, but not in Experiment 2. For reasons of space, we only present the stimuli of Experiment 1:

**Table:**

(1) Zhe-zhi xiaogou ya, zai da xiaomao.	(SVO topicalization)
‘As for this dog, (it) is hitting the cat.’	
(2) Zhe-ge haizi ya, waipo zai hua.	(OSV topicalization)
‘As for this child, the grandma is drawing (him).’	
(3) Zhe-ge nanhai, zai kan shu.	(filler)
‘As for this boy, (he) is reading a book.’	
(4) Zhe-zhi xiaogou ya, zai kan xiaomao.	(SVO topicalization)
‘As for this dog, (it) is looking at the cat.’	
(5) Zhe-ge nanhai ya, xiaogou zai tui.	(OSV topicalization)
‘As for this boy, the dog is pushing (him).’	
(6) Zhe-ge nanhai, zai qi zixingche.	(filler)
‘As for this boy, (he) is riding a bike.’	
(7) Zhe-ge nanhai ya, daxiang zai gai.	(OSV topicalization)
‘As for this boy, the elephant is covering (him).’	
(8) Zhe-zhi xiaomao ya, xiaogou zai yao.	(OSV topicalization)
‘As for this cat, the dog is biting (it).’	
(9) Zhe-ge nühai, zai chuan qünzi.	(filler)
‘As for this girl, (she) is wearing a skirt.’	
(10) Zhe-ge nanhai ya, zai bang daxiang.	(SVO topicalization)
‘As for this boy, (he) is helping the elephant.’	
(11) Zhe-zhi houzi ya, zai zhui xiaogou.	(SVO topicalization)
‘As for this monkey, (it) is chasing the dog.’	
(12) Zhe-ge yeye, zai jiao hua.	(filler)
‘As for this grandpa, (he) is watering flowers.’	
(13) Zhe-zhi houzi ya, laoshu zai kan.	(OSV topicalization)
‘As for this monkey, the mouse is looking at (it).’	
(14) Zhe-ge nanhai, zai ti qiu.	(filler)
‘As for this boy, (he) is playing football.’	
(15) Zhe-zhi qingwa ya, zai hua gongzhu.	(SVO topicalization)
‘As for this frog, (it) is drawing the princess.’	
(16) Zhe-ge nühai, zai cui lazhu.	(filler)
‘As for this girl, (she) is blowing out candles.’	
(17) Zhe-zhi qingwa ya, laoshu zai da.	(OSV topicalization)
‘As for this frog, the mouse is hitting (it).’	
(18) Zhe-zhi xiaomao ya, zai tui daxiang.	(SVO topicalization)
‘As for this cat, (it) is pushing the elephant.’	
(19) Zhe-ge nühai, zai tiao sheng.	(filler)
‘As for this girl, (she) is jumping rope.’	
(20) Zhe-ge nühai ya, zai gai waipo.	(SVO topicalization)
‘As for this girl, (she) is covering the grandma.’	
(21) Zhe-zhi laoshu ya, daxiang zai zhui.	(OSV topicalization)
‘As for this mouse, the elephant is chasing (it).’	
(22) Zhe-ge nanhai, zai zhai yingtao.	(filler)
‘As for this boy, (he) is picking up cherries.’	
(23) Zhe-ge nühai ya, waipo zai bang.	(OSV topicalization)
‘As for this girl, the grandma is helping (her).’	
(24) Zhe-zhi laoshu ya, zai yao xiaomao.	(SVO topicalization)
‘As for this mouse, (it) is biting the cat.	
